# Lyme Meningoencephalitis Masquerading as Normal Pressure Hydrocephalus

**DOI:** 10.7759/cureus.2417

**Published:** 2018-04-03

**Authors:** Aakash Desai, Gaurav Manek, Anand M Krishnan, Corina Iorgoveanu, Ahmed Zaghloul

**Affiliations:** 1 Internal Medicine, University of Connecticut Health Center , Farmington, USA; 2 Internal Medicine, University of Connecticut Health Center, Farmington, USA

**Keywords:** lyme disease, meningoencephalitis, normal pressure hydrocephalus

## Abstract

Lyme disease is a tick-borne illness caused primarily by the spirochete *Borrelia burgdorferi*. The disease is most prevalent in forested areas endemic for Ixodes tick, which transmits the spirochete. Here, we describe a case of Lyme meningoencephalitis masquerading as normal pressure hydrocephalus (NPH) which initially presented with urinary incontinence, gait instability, and neurological decline. Due to its non-specific symptoms and low incidence, Lyme meningoencephalitis causing NPH like syndrome poses a diagnostic conundrum for clinicians. Awareness of this disease entity is key for prompt diagnosis and treatment.

## Introduction

Lyme disease is a tick-borne illness caused primarily by the spirochete *Borrelia burgdorferi*. The disease is most prevalent in forested areas endemic for Ixodes tick, which transmits the spirochete. Here, we describe a case of Lyme meningoencephalitis masquerading as normal pressure hydrocephalus (NPH) which initially presented with urinary incontinence, gait instability, and neurological decline. Due to its non-specific symptoms and low incidence, Lyme meningoencephalitis causing NPH like syndrome poses a diagnostic conundrum for clinicians. Awareness of this disease entity is key for prompt diagnosis and treatment.

## Case presentation

**Figure 1:**
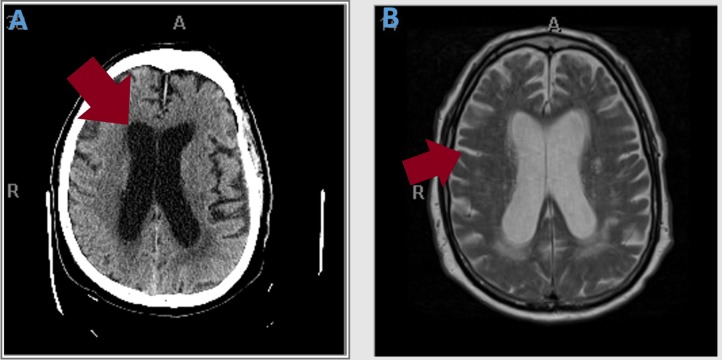
A) Computed tomography scan of the head without contrast (arrow shows ventriculomegaly) and B) Magnetic resonance imaging brain with contrast (arrow shows cerbral atrophy and loss of sulci and gyri)

**Figure 2:**
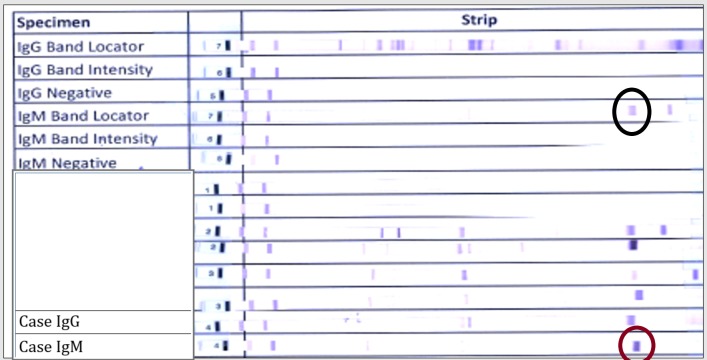
Lyme disease serum antibody Western blot (black circle: position of IgM bands in control; red circle: presence of IgM bands in the case)

An 87-year-old male was being evaluated for urinary incontinence and recent onset of unsteady gait by his geriatrician. Due to progressive weakness with a significant neurological decline to the point that he was unable to get out of the bed, his family decided to bring him to the hospital. At the time of initial evaluation, he had bilateral hand tremors, dysmetria, dysdiadokokinesia and memory impairment related to place and time. He was also diagnosed with community acquired pneumonia and was treated with ceftriaxone. A computed tomography (CT) scan of the head revealed an increase in the size of the third and lateral ventricles suggesting communicating hydrocephalus superimposed on cerebral atrophy secondary to chronic lacunar infarcts (Figure 1A). Magnetic resonance imaging (MRI) revealed that the ventriculomegaly was more likely due to cerebral atrophy than balanced hydrocephalus (Figure 1B). Due to the presence of triad symptoms of urinary incontinence, altered mental status, and unsteady gait, a presumptive diagnosis of NPH was made and a large volume spinal tap was performed; this led to modest improvement in his gait. However, on spinal fluid analysis, lymphocytic pleocytosis was noted. Further workup for possible viral, fungal or inadequately treated bacterial meningoencephalitis was pursued. As he had recently been outdoors for a fourth of July picnic, Lyme serologies were added to his workup (Figure 2). Empiric treatment was initiated with acyclovir, vancomycin, ampicillin-sulbactam. Despite this, his condition continued to worsen, with increasing tremulousness, worsening dysmetria, and more impaired cognition. Serologies were indicative of an early Lyme infection. Given his clinical deterioration, we decided to continue the ceftriaxone 2 grams daily to empirically treat possible Lyme meningoencephalitis. He improved dramatically over the following two days. Lyme Immunoglobulin M (IgM) was positive in the cerebrospinal fluid (CSF) confirming the diagnosis of Lyme meningoencephalitis. He continued to improve and was discharged on ceftriaxone to complete a 4-week course to a short-term rehabilitation facility.

## Discussion

Lyme disease is a tick-borne illness caused primarily by the spirochete *Borrelia burgdorferi*. The disease is most prevalent in forested areas endemic for Ixodes tick, which transmits the spirochete [1]. Classically, clinical presentation of Lyme disease is classified into three stages: early localized disease (includes the classic erythema migrans), early disseminated disease (includes carditis, neurological manifestations, musculoskeletal, skin involvement and lymphadenopathy), and late disseminated disease (neurological disease including peripheral neuropathy/ encephalomyelitis) [2]. Diagnosis requires a detailed travel and activity history and a high index of suspicion, since laboratory findings may be unrevealing, although high sedimentation rates and serum transaminases can be found. Serology is the most important part of Lyme disease diagnosis. A two-tier algorithm is used for diagnosis, which involves enzyme linked immunosorbent assay (ELISA) followed by Western blot confirmation if the screening ELISA is positive [3]. Although classically neurological manifestations of Lyme occur in later stages of the disease, acute neuroborreliosis has been described as a severe complication of Lyme borreliosis [4]. Another impediment to correct diagnosis of neuroborreliosis is the wide spectrum of presentations ranging from stroke [5] to a syndrome similar to Alzheimer’s disease [4]. A study by Schwenkenbecher et al. [6] which studied the various presentations of neuroborreliosis leading to hospitalization demonstrated that cranial nerve palsy was the most frequent deficit (50%) followed by painful radiculitis (25%), encephalitis (12%), myelitis (7%), and meningitis/headache (6%). We describe a case which illustrates the potential for Lyme meningoencephalitis, also termed as "neuroborreliosis", presenting with atypical features mimicking NPH. Danek et al. [7] first described the syndrome of NPH in a patient with Lyme neuroborreliosis. Recently, Aboul-Enein et al. [8] and Topakian et al. [9] have also demonstrated a similar syndrome which shows a dramatic response to antibiotic treatment. It has been hypothesized that neuroborreliosis may cause secondary NPH by interfering with subarachnoid CSF flow due to inflammatory response to Lyme antigens [7]. Thus, treatment of the infection with adequate antibiotic therapy helps in reducing any subsequent inflammation leading to proper drainage of the CSF.

## Conclusions

In conclusion, although the nervous system involvement in Lyme disease, as seen in our patient, is more commonly noted later during the disease, it can also present earlier in an endemic setting. It is important to recognize the presence of a NPH like syndrome as a subset of Lyme neuroborreliosis. Having a high index of suspicion aids in the early recognition of this syndrome, which is critical to the institution of appropriate therapy and prevention of long-term neurological complications from unrecognized Lyme disease.
